# The feasibility of a Comprehensive Resilience-building psychosocial Intervention (CREST) for people with dementia in the community: protocol for a non-randomised feasibility study

**DOI:** 10.1186/s40814-020-00701-2

**Published:** 2020-11-16

**Authors:** Dympna Casey, Niamh Gallagher, Declan Devane, Bob Woods, Kathy Murphy, Siobhán Smyth, John Newell, Andrew W. Murphy, Charlotte Clarke, Tony Foley, Fergus Timmons, Rose-Marie Dröes, Martin O’Halloran, Gill Windle, Kate Irving Lupton, Christine Domegan, Eamon O’Shea, Pat Dolan, Priscilla Doyle

**Affiliations:** 1 School of Nursing & Midwifery, National University of Ireland Galway, Aras Moyola, Galway, Ireland; 2 Dementia Services Development Centre, Bangor University, Bangor, Wales; 3 School of Mathematics, Statistics & Applied Mathematics, National University of Ireland Galway, Galway, Ireland; 4 Department of General Practice, School of Medicine, National University of Ireland Galway, Galway, Ireland; 5 Social Sciences and Health, Durham University, Durham, UK; 6 Department of General Practice, University College Cork, Cork, Ireland; 7 The Alzheimer Society of Ireland, Temple Road, Blackrock, Co. Dublin Ireland; 8 Department of Psychiatry, VU University Medical Center, A.J. Ernststraat 1187 (kamer D0.03), Amsterdam, The Netherlands; 9 College of Engineering & Informatics, National University of Ireland Galway, Galway, Ireland; 10 School of Health Sciences, Bangor University, Bangor, Wales; 11 School of Nursing, Psychotherapy and Community Health, Dublin City University, Dublin, Ireland; 12 J.E. Cairnes School of Business & Economics, National University of Ireland Galway, Galway, Ireland; 13 Centre for Economic & Social Research on Dementia, ILAS Building, National University of Ireland, Galway, Ireland; 14 UNESCO Child and Family Research Centre, School of Political Science and Sociology, National University of Ireland, Galway, Ireland

**Keywords:** People with dementia, Caregivers, Resilience, Psychosocial interventions, Non-randomised feasibility study

## Abstract

**Background:**

A dementia diagnosis can prevent people from participating in society, leading to a further decline in cognitive, social and physical health. However, it may be possible for people with dementia to continue to live meaningful lives and continue to participate actively in society if a supportive psychosocial environment exists. Resilience theory, which focuses on strengthening personal attributes and external assets in the face of serious challenges, may provide a scaffold on which an inclusive multifaceted psychosocial supportive environment can be built. This protocol paper describes a study to determine the feasibility of conducting a multifaceted complex resilience building psychosocial intervention for people with dementia and their caregivers living in the community.

**Methods:**

This is a non-randomised feasibility study. Ten participants with dementia and their primary caregivers living in the community will be recruited and receive the CREST intervention. The intervention provides (a) a 7-week cognitive stimulation programme followed by an 8-week physical exercise programme for people with dementia and (b) a 6-week educational programme for caregivers. Members of the wider community will be invited to a dementia awareness programme and GP practices to a dementia training workshop. Trained professionals will deliver all intervention components. Outcomes will assess the feasibility and acceptability of all study processes. The feasibility and acceptability of a range of outcomes to be collected in a future definitive trial, including economic measurements, will also be explored. Finally, social marketing will be used to map a route toward stigma change in dementia for use in a subsequent trial. Quantitative feasibility outcome assessments will be completed at baseline and after completion of the 15-week intervention while qualitative data will be collected at recruitment, baseline, during and post-intervention delivery.

**Conclusion:**

This feasibility study will provide evidence regarding the feasibility and acceptability of a comprehensive multifaceted psychosocial intervention programme for people with dementia and their caregivers (CREST). The results will be used to inform the development and implementation of a subsequent RCT, should the findings support feasibility.

**Trial registration:**

ISRCTN25294519 Retrospectively registered 07.10.2019

**Supplementary information:**

**Supplementary information** accompanies this paper at 10.1186/s40814-020-00701-2.

## Background

Dementia is a progressive neurocognitive disorder [1] characterised by memory and cognitive impairment as well as behaviour changes, all of which impact on the ability of the person with dementia to undertake activities of daily living [2]. It is estimated that there will be 152 million people with dementia worldwide in 2050 [2]. Therefore, dementia is a major contributor to the global burden of disease with a yearly estimated cost of US$818 billion dollars [3], projected to rise to $2 trillion by 2030 [2, 4–6]. Furthermore, almost 85% of costs are related to family and social care costs [5]. It is not surprising therefore that dementia is considered one of the biggest health, societal and economic challenges of the twenty-first century [2, 5–7].

A dementia diagnosis may also exacerbate social inequality and discrimination [8]. This diagnosis can lead to marginalisation within the health services [9] and may prevent people with dementia from participating in society [8, 10]. However, it may be possible for people with dementia to continue to live a meaningful life, retain many abilities and continue to actively participate in society, if there is a supportive psychosocial environment that maximises functioning and social connectedness [5, 11, 12]. Resilience theory is promising as it focuses upon strengthening people’s resources in the face of serious challenges and difficulties [13], and may, by enhancing people’s strengths and valued relationships, provide a scaffold upon which an inclusive supportive psychosocial environment can be built.

Resilience, defined as a “dynamic and amendable process” [14], encompasses positive adaptation within the context of major adversity. It focuses on modifiable intra-personal skills and protective factors aimed at increasing a person’s ability to remain psychologically, socially and physically healthy, or ‘resilient’, in the face of adversity.

Windle [15] describes resilience as a ‘multilevel construct’ and a dynamic lifetime process. She presents a resilience framework that focuses on individual, community and societal components that impact the resilience of older people with dementia and their caregivers. Similarly, Harris [16] recommends that resilience-boosting interventions should be multifaceted, targeting the individual, family and community. She outlines that at the individual level, resilience promoting factors include the person with dementia accepting changes in self; an emphasis on nurturing the person's remaining skills; focusing on the positives and what is retained; recognising the multiple ways in which a person with dementia can make a meaningful contribution to family, friends and/or the community. At the level of family and community, resilience-promoting factors include having a supportive doctor; having connections to meaningful community groups and activities, and having a supportive and positive social environment that promotes dignity and respect and reasonable independence. Examining the assets and protective factors both internal and external to the person with dementia is considered central to effective resilience building in people with dementia [13, 14, 17, 18]. Many other writers also endorse the position that psychosocial resilience- building interventions need to be multi-faceted, focusing on both the personal attributes and external assets of people with dementia [15, 16, 19–22].

However, what precisely the preferred components of a multifaceted psychosocial resilience-building intervention might be for people living with dementia is relatively unknown. To address this deficit, we interviewed a sample of people with mild-moderate dementia (*n* = 6) [23], carers (*n* = 28) [24] and 12 informal caregivers/care partners [25]. Analysis of these interviews identified five areas of importance to resilience building: having personal control and a ‘fighting spirit’, strong family relationships, staying connected to communities, undertaking physical activity and raising awareness and tackling negative attitudes through dementia education.

It is clear from the interviews with people with dementia, caregivers and the literature that psychosocial resilience building interventions need to be multi-faceted, focusing on both the personal attributes and external assets of people with dementia. McDermott et al. [26] carried out a systematic review of the effectiveness of a wide range of psychosocial interventions to meet the physical, cognitive, psychological and social needs of people with dementia. The evidence drawn from 22 reviews evaluating 197 studies indicated that both group cognitive stimulation therapy and physical exercise have recognisable benefits for people with dementia.

Utilising this evidence and drawing on the expertise of the research team, we identified three successful psychosocial interventions, namely, cognitive stimulation, physical exercise and education, which we hypothesised could be combined to create a novel multifaceted psychosocial resilience-building intervention simultaneously targeting the personal attributes and external assets of people with dementia.

### Cognitive stimulation therapy

Cognitive stimulation therapy (CST) is a non-pharmacological psychosocial intervention aimed at building both internal and external assets of people with mild to moderate dementia [27]. It involves engaging the person with dementia in a range of activities, typically delivered in a group setting, aimed at improving cognitive and social functioning [28]. Many studies reveal that CST has a positive impact on cognitive functioning, and quality of life of people with dementia [29, 30] as well as having positive outcomes on social interaction and communication [29]. Furthermore, a Cochrane systematic review on CST, which included 15 randomised controlled trials with a total of 718 participants, found that people with dementia receiving CST had significantly improved cognition, communication and quality of life compared to those receiving usual care or an alternative activity [26, 27]. CST is also recommended for people with mild to moderate dementia [10, 31]. CST therefore has the potential to build the internal and external assets of people with dementia.

### Exercise

Physical exercise is important for cognition and brain health because it impacts on other modifiable risks factors such as obesity, hypertension, cardiovascular and metabolic risk factors [5]. Studies also indicate that exercise may decrease inflammation and cholesterol and promote beneficial growth factors in the brain [32, 33]. Although randomised trials have not yet demonstrated that exercise delays cognitive decline or dementia, observational studies do demonstrate an inverse relationship between exercise and risk of dementia [5]. In particular, high-intensity multi-component exercises, including walking, stretching and other strength exercises seemed to be most beneficial [26]. Furthermore, group activities and group exercise interventions with a strong social element have been found to be beneficial and promote social connectedness [26, 34] and life satisfaction, and reduce aggression and night-time restlessness [35, 36]. Likewise, Junge et al. [37] in their systematic mixed studies review exploring the effect and importance of physical activity on behavioural and physiological symptoms of dementia found that of key importance was the ‘socially rewarding’ aspect of the group-based physical activities.

### Dementia education

Dementia education for informal caregivers, health care professionals and members of the public has been identified as key to enhancing the quality of life of people with dementia [38].

Informal caregivers provide most of the care for people with dementia living at home [39]. These caregivers are therefore a crucial resource/external asset for people with dementia. However, they are often unsupported in their caregiving role, which may result in the person with dementia being placed in residential care prematurely [39]. Dickinson et al. [22] undertook a systematic review of 31 systematic reviews of psychosocial interventions for informal caregivers of people with dementia. They found that effective educational interventions included presenting structured information about dementia and caregiving issues, actively involving caregivers, and delivering the educational programme in a support group format. Other studies reveal that psychosocial educational interventions which include information on how dementia affects the brain, different types of dementia, skills to effectively communicate with people with dementia, how to manage behavioural symptoms, as well as information on local resources have been found to be effective in helping caregivers to more effectively work with, support and care for people with dementia [40–42] and lead to more person- centered attitudes, empathy and improved sense of competence.

Within the community, the general practitioner (GP) is the first health care professional to be consulted when dementia is suspected by the person themselves or by a family member [43]. As outlined by Harris [16], a supportive doctor is an important community level resilience- building resource for people with dementia. However, GPs acknowledge that the complexities associated with the diagnosis and management of dementia is challenging and recognise a need for further education and training [44]. Providing educational support to GP’s, in particular interactive small group work [45], may enable them to provide optimal care and promote resilience in people with dementia.

Finally, the broader community and members of the public are key resilience promoting factors for people with dementia, influencing the extent to which the person with dementia can continually function and remain in their own environment [38]. However, a lack of understanding of dementia can lead to fear and stigmatisation, which contributes to the social isolation of people with dementia [46]. Dementia awareness raising interventions can help combat stigma by tackling public perceptions [47]. In particular, public education is considered a key strategy in reducing the stigma associated with dementia [9, 46–55]. The focus is on reducing the knowledge gap [9] and replacing myths with accurate information [9, 49, 50, 54]. Social contact in particular is considered the most effective way of reducing stigma and improving attitudes toward dementia [56] as it reduces anxiety about contact and promotes empathy [53]. To be effective, this social contact ideally needs to be targeted, local, credible and sustainable [57], thereby building the external assets of people with dementia. Enhancing and extending the social networks of the person with dementia creates new connections and strengthens relationships [58], thereby building their external resources/assets.

In summary, psychosocial interventions utilising either CST, or physical exercise or education have the capacity to strengthen either personal attributes or external assets of people with dementia. However, multifaceted psychosocial interventions are required to build resilience, which simultaneously target the personal and external assets of people with dementia. We therefore combined CST, exercise and education to create a novel, multifaceted, psychosocial resilience building intervention for use in a future definitive RCT.

## Methods

### Study aim

This study will assess the feasibility of, and inform the optimal design of, a future proposed definitive randomised trial to examine the effectiveness of the CREST intervention for people with dementia and their caregivers living in the community.

### Objectives

1. To assess the feasibility and acceptability of the proposed CREST intervention to participants.
2. To test the feasibility and acceptability of a proposed future definitive trial.

### Design

**Fig. 1:**
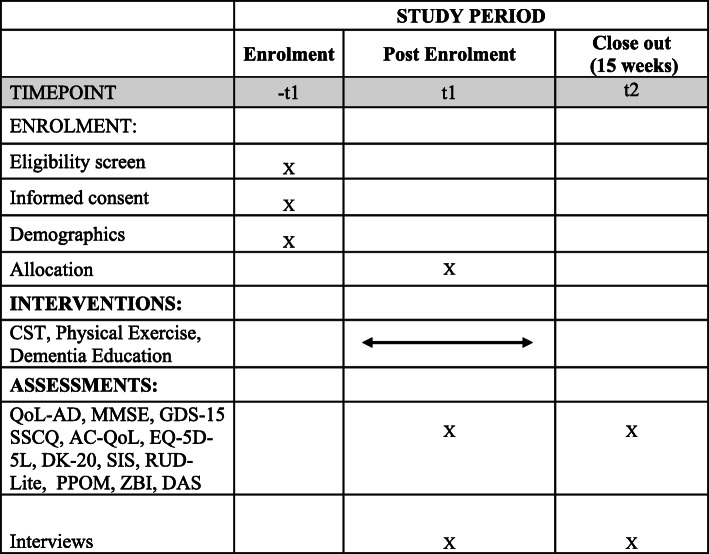
Schedule of enrolment, interventions and assessments as per SPIRIT 2013

This study will use a non-randomised feasibility design and will follow the SPIRIT 2013 [59] guidelines and reporting template (Additional file 1). A schedule for enrolment, interventions and assessments is displayed in Fig. 1.

### Participants

#### People with dementia

Inclusion criteria

- >60 years of age
- Have either
  i. A formal diagnosis of mild to moderate dementia
  ii. Are prescribed dementia medications
  iii. Or their GP believes the person has memory problems and the person has a provisional diagnosis of dementia based on the DSM-IV criteria
- And are
- Living in the community
- Willing and capable of undertaking the exercise component of the intervention
- Able to speak and read English
- Able to give informed consent
- Primary care giver is also willing to take part in the study

#### Caregivers of people with dementia

Inclusion criteria

- Primary caregiver of a person with dementia who has also agreed to participate in the study
- Does not have a diagnosis of dementia
- Living in the community
- Willing and able to take part in the 6 weeks caregiver education programme
- Able to speak and read English
- Able to give informed consent

### Intervention

**Fig. 2:**
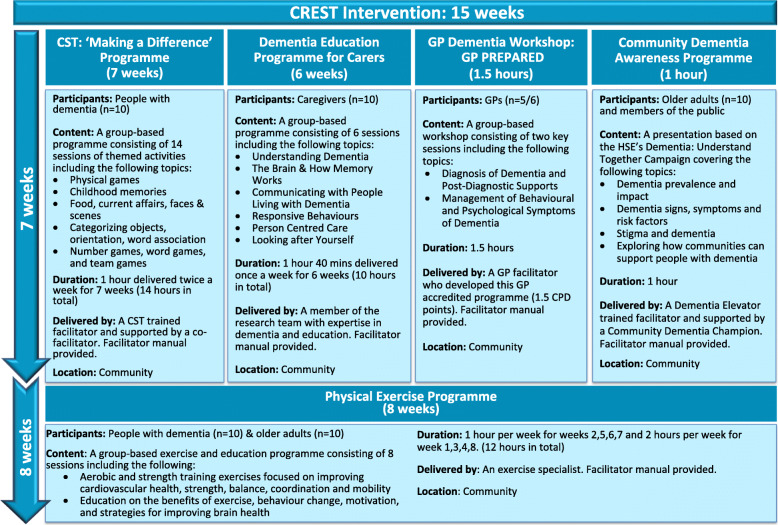
Overview of the CREST intervention components

The CREST intervention is a multi-level complex psychosocial intervention of 15 weeks duration. It consists of three key interrelated components targeted at people with dementia: their informal caregivers, GPs and the wider community. CREST consists of three components, cognitive stimulation therapy, physical exercise and dementia education. An overview of the intervention components, their duration, content, number and targeted participants is presented in Fig. 2.

1. *Cognitive stimulation therapy (CST)*

In this feasibility study, the ‘Making a difference’ CST programme developed by Spector et al. [12] will be used. This programme consists of 14 sessions delivered over the first 7 weeks of the intervention. In CREST, participants with dementia will attend two 1 h sessions per week and each session will include approximately five participants. Sessions will include a focus on the use of reminiscence and providing triggers to aid recall, stimulating language and executive functioning and being person-centred.

A health care professional trained to deliver the programme using the CST manual and supported by a co-facilitator will facilitate each session. The role of the co-facilitator will be to help set up the class and support, prompt and encourage participants to adhere to the programme.

2) *Physical exercise programme*

An exercise programme, based on the PRINCE structured exercise programme [60] modified to meet the needs of people with dementia, will be used. This exercise programme, delivered over 8 weeks (after the CST programme), is a total of 12 h in duration: 1 h session a week for 4 weeks and 2 h sessions a week for 4 weeks (see Fig. 2). The programme is designed to be delivered in a group class and consists of a combination of aerobic and strength training exercises appropriate for people with dementia and education content specific to exercising. A physical exercise specialist supported by an exercise manual will facilitate the programme. Older adults from the community will support participants with dementia to undertake the exercises. The older adults will be invited to assist in the exercise sessions if they are:

- > 60 years of age
- Living in the community
- Do not have a diagnosis of dementia
- Willing and capable of undertaking the exercise programme
- Willing to support people with dementia to undertake the exercise programme
- Able to speak in and read English
- Able to give informed consent

These older adults, who form part of the intervention, will assist people with dementia by prompting them through their exercises and providing support, encouragement as well as social interaction and engagement. They will also assist people with dementia to record and track their exercise progress.

The exercise specialist will assess the progress, duration and intensity of the programme for people with dementia throughout the intervention, in order to increase participants’ exercise capacity gradually. In week 1, participants with dementia will be asked to identify specific exercise-related goals they wish to achieve by the end of the programme. These will be reviewed in weeks 4 and 8. The exercise programme will begin with 10 × 3-min circuits. Participants with dementia will start at a station and rotate to the next station until all 10 circuits are completed. Each person with dementia will undertake the exercise at each station 8–10 times with three repetitions. At the end of 3 min, the person will record their exercises and move to the next station. In week 3, the exercise specialist will assess participants and prescribe weights for strengthening. Participants will be encouraged to complete some of the exercises at home and record these in a home exercise diary. In addition, each person with dementia will be asked to wear a Fitbit wearable activity tracker to measure changes in exercise activity and sleep quality before, during and after the physical exercise programme. Researchers will assist participants to set up their Fitbit and how to access their exercise performance on the tracker. The feasibility and acceptability of wearing this device as well as the extent to which the Fitbit acted as an exercise motivational tool will be captured via qualitative interviews.

3) *Dementia education programme*

This programme consists of three elements:

- Community dementia awareness programme

This programme is targeted at members of the public in the wider community. It is designed to provide information on dementia, create positive action for dementia and tackle negative attitudes toward dementia. This programme, based on the Irish Health Service Executive’s [61] *Dementia: Understand Together* campaign will be 1 h in duration. It will be delivered and co-facilitated by dementia champions trained to deliver the programme. The programme will include information about dementia prevalence and impact, signs and symptoms, risk factors, and stigma and explores how communities can support the health and well-being of a person with dementia and their family.

(b) Caregiver programme

This interactive and participatory 10-h (1 h 40 min a week) educational programme will be delivered over the first 6 weeks (coinciding with the CST programme) (Fig. 2). It will be facilitated by members of the research team with expertise in dementia, nursing and education. This programme is based on the DARES structured education programme [62] modified to meet the needs of informal caregivers of people with dementia and informed by The Alzheimer Society of Ireland’s Family Carer Training programme [63]. The aim of this programme is to develop the caregivers’ knowledge and skills regarding dementia to enable them to respond more confidently to the needs of the person with dementia, provide them with ‘me time’ and an opportunity to focus on their own health needs and to meet other caregivers and share experiences. An overview of the content of the programme is outlined in Fig. 2.

(c) GP educational workshop

This workshop was developed by the PREPARED team (Primary Care Education, Pathways and Research of Dementia) http://dementiapathways.ie/_filecache/d43/336/634-gp-facilitator-workshop-guide.pdf. This evidence-based 1.5-h interactive, Continuous Professional Development (CPD)-accredited dementia workshop aims to support GPs and primary care teams in their delivery of integrated dementia care. Developed following an educational needs analysis, the workshop focuses on dementia diagnosis, post-diagnostic care and management of the behavioural and psychological symptoms of dementia. The peer-facilitated workshop will be delivered by a GP trained to deliver the programme.

### Control group

This will be a single arm feasibility study and will not have a comparison group.

### Feasibility study outcomes

Given the aim of the study, outcomes focus on feasibility and include:

1. Number of participants (people with dementia and caregivers) who are screened, judged eligible and agree to take part in the study.
2. Identification of optimal strategy for recruitment of participants for future definitive trial.
3. Identification of barriers and enablers to stigma change in dementia.
4. Willingness of key gatekeepers, for example, GPs Local Alzheimer Cafés and Western Alzheimer groups, to recruit participants (people with dementia and caregivers).
5. Feasibility and acceptability of the intervention content, delivery and fidelity assessments.
6. Follow-up rates, outcome completion and adherence/compliance rates.
7. Reasons for non-recruitment, non-adherence or attrition.
8. Acceptability of the recruitment process, assessments, data collection tools, intervention content and delivery to participants.
9. Baseline score and variability of secondary outcome measures among participants to inform sample size estimates for a future definitive trial.
10. Evaluation of cost analyses process.

### Embedded qualitative study

#### Design

A qualitative descriptive approach based on the work of Sandelowski [64] will be used to capture key stakeholders views as to the acceptability and feasibility of respective study procedures, including the recruitment process, intervention content and delivery, data collection methods, as well as on the factors that facilitated or hindered engagement and adherence. This data will help identify any feasibility issues, which may need to be addressed to inform the design of a subsequent RCT.

A sample of participants with dementia (*n* = 5) and caregivers (*n* = 5) will be interviewed during the delivery of the CST programme and caregiver programme respectively (weeks 4–5). In addition, a sample of participants with dementia (*n* = 5) will be interviewed during the delivery of the physical exercise programme (weeks 11-12). These interviews will focus on capturing the ongoing perceptions and experiences of participants as they progress through the intervention. All participants will be interviewed post-intervention. Interviewing some participants during and post- intervention will provide information as to how the intervention is progressing and why things may have changed over time as they progressed through the intervention.

The CORTE interviewing framework developed to maximise the meaningful involvement of people with dementia will be used [65]. This guide consists of gaining **CO**nsent, maximizing **R**esponses, **T**elling the story, and **E**nding on a positive note. All facilitators/co-facilitators (*n* =9) as well as the older adults (*n* = 10) supporting the delivery of the exercise component will also be interviewed to capture their experiences of delivering and facilitating the intervention and identify any potential areas for improvement. In total, 54 interviews will be undertaken. Table 1 presents a summary overview of participants who will be interviewed, and the time point for these interviews.

**Table 1:** Overview of the qualitative data collection process

Participants	Number interviewed Week 4-5	Number interviewed weeks 11-12	Number interviewed post-intervention Weeks 16–19	Total number of interviews
**People with dementia**	5 (CST)	5 (Physical Exercise)	10	20
**Caregivers**	5	0	10	15
**Facilitators and co-facilitators**			4 (CST) 2 (Physical Exercise) 2 (Community Dementia Awareness programme) 1 (GP Prepared programme)	9
**Older adults**			10	10
**Totals**	10	5	39	54

### Main trial outcome measurements

Although this is a feasibility study, we will explore the feasibility and acceptability of a range of secondary outcome measurement tools for use in a future definitive trial. A summary of these measures and the participant from which the outcome will be collected and recorded is presented in Table 2, while Table 3 presents a summary overview of how the pilot may inform a future definitive trial.

**Table 2:** Summary overview of secondary outcome measures for future definitive trial

**People with dementia: Data collected at baseline and post 15-week intervention**	
**Quality of Life-Alzheimer’s Disease (QoL-AD)** [66]. The QoL-AD is a brief, 13-item measure designed specifically to obtain a rating of the person’s quality of life from either the person living with dementia and/or the caregiver [66]. For people with dementia, the questionnaire is completed during an interview that usually takes 10–15 min. The QoL-AD has very good psychometric properties and can be completed with people with a wide range of severity of dementia [67]. Internal consistency is good with a Cronbach’s alpha coefficient of 0.82 [67]. The scale has good content validity, and it also correlates well with the Dementia Quality of Life scale (0.69) and with the Euroqol-5D scale (0.54), indicating good criterion concurrent validity [67].	
**Mini-Mental State Examination (MMSE)** [68]. The MMSE consists of questions covering 11 domains. It is a simplified, scored form of the cognitive mental status examination, which assesses the cognitive aspects of mental functions.	
**The Geriatric Depression Scale–Short Form (GDS-15) **[69]. This is a 15-item self-completed questionnaire or administered interview used to measure depression in older adults and takes 5–7 min to complete. Mitchell et al. [70] completed a meta-analysis on the diagnostic accuracy, clinical utility and added value of the GDS in primary care. They concluded that the GDS-15 has acceptable sensitivity and specificity (81.3 and 78.4%, respectively), “good” clinical utility for screening and should be used in the diagnosis of late-life depression in primary care.	
**Stigma Impact Scale (SIS)** [71]. The SIS has 24 items and is completed by people with dementia to measure the perceived stigma inherent in progressive neurological diseases. The SIS can be administered via interview or self-completed and on average takes 10 min to complete. The internal consistency has a Cronbach’s alpha of 0.89, indicating good reliability [71]. Construct validity scores range from 0.44 to 0.84 [71].	
**Positive Psychology Outcome Measure (PPOM)** [72]. The PPOM is used to measure hope and resilience in people with dementia. It consists of 16 items designed for either self-completion by participants or completion via an interview and takes 5–10 min to complete. The internal consistency has a Cronbach’s alpha of 0.94, indicating excellent reliability [72]. The PPOM remained moderately stable over a 1 week period (ICC: 880), and factor analyses indicated a two-factor structure solution with acceptable fit indices [72]. The PPOM was developed from the Resilience Scale of Wagnild and Young [73], an instrument which scored highly in Windle et al. [74] review as a good measure of resilience.	
**EQ-5D-5L** [75]. The EQ-5D-5L is a 6-item standardized questionnaire that measures self-reported generic health status. The scoring system translates the quality of life scores into an economic value. Domain responses and utility scores have good test-retest reliability (ICC, 0.777; agreement, 76.4–98.1%) [76]. Scores of domains of the EQ-5D-5L correlated significantly (*r*, 0.57–0.74) with the scores of the SRS-22r domains, supporting construct validity [76].	
**Fitbit** The Fitbit will allow participants’ sleep patterns and exercise activity to be monitored. Each Fitbit will be synced to a mobile device or PC. The Fitabase data management platform will be used to extract and aggregate participant data to facilitate analysis.	
**Caregivers: Data collected at baseline and post 15-week intervention**	
**Zarit Burden Interview (ZBI)** [77]. The ZBI is a 22-item self-completed questionnaire or administered interview used to assess the level of burden experienced by the primary caregivers of older people with dementia and people with disabilities. The ZBI takes on average 10 minutes to complete. It is a valid and reliable instrument with a Cronbach’s alpha value of 0.93; intra-class correlation coefficient for the test-retest reliability of 0.89 (*n* = 149) [78].	
**Short Sense of Competence Questionnaire (SSCQ**) [79]. The SSCQ is a 7-item self-completed questionnaire or administered interview, which measures the sense of competence the caregiver of a person with dementia has. The SSCQ takes on average 5 min to complete. It is a valid and reliable instrument with a Cronbach’s alpha value of 0.76 [79].	
**Dementia Knowledge 20 (DK-20)** [80]. The DK-20 is a 20-item self-administered questionnaire, which measures the knowledge people have regarding dementia. It is a 20-item self-administered questionnaire that takes on average 15 min to complete. Convergent validity reveals significant correlation between the DK-20 scale and the ADQ calculated using Spearman’s one-tailed test, *r*(175) = 0.36, *p* < 0.001 [80]. Test-retest reliability for the total scale is ICC = 0.73, *p* < 0.001, which indicated substantial reliability of the scale [80].	
**Resource Utilization in Dementia-Lite (RUD-Lite)** [81]. The RUD-lite questionnaire consists of 25 items, and measures healthcare resource utilization among older adults with dementia and their caregivers, and time spent on formal and informal care by caregivers. The questionnaire is completed by the primary caregiver of the person with dementia and takes on average 15 minutes to complete. Wimo et al. [82] found the estimations provided by primary caregivers completing the RUD-lite to be accurate. The agreement between diaries and recall estimates was high for personal ADL (intra-class correlation (ICC) 0.93), supervision (ICC 0.87) and total time (ICC 0.91) and lower but acceptable for instrumental ADL (ICC 0.75) [82].	
**Adult carer quality of life (AC-QoL)** [83]. The AC-QoL is a 40-item questionnaire, which measures the overall quality of life of adult caregivers over 8 domains: support for caring, caring choice, caring stress, money matters, personal growth, sense of value, ability to care, and caregiver satisfaction. The questionnaire takes 10 minutes to complete. Construct validity using exploratory factor analysis was conducted by Negri et al. [84] using the Kaiser–Meyer–Olkin (KMO) measure with a total score of 0.90, and all KMO values for single items were higher than 0.70, thus above the acceptability limit of 0.50 [84]. Cronbach alpha scores for the eight subscales range from 0.79 to 0.90, showing acceptable to excellent levels of reliability; alpha coefficient for the AC-QoL summed score was also excellent (0.93) [84].	
**Older people: Data collected at baseline and post 15-week intervention**	
**Dementia Attitudes Scale (DAS)** [85]. The dementia attitude’s scale is a 20-item questionnaire that measures attitudes towards dementia of members of the public, working professionals, and students. The DAS measures attitudes towards dementia of members of the public, working professionals, and students. DAS correlates significantly with scales that measure ageism and attitudes toward disabilities and has a Cronbach’s alphas ranging from 0.83 to 0.85 [85].	
**Dementia Knowledge-20 (DK-20) **[80].

**Table 3:** Summary overview of how the pilot may inform a future definitive trial

Pilot study-feasibility and acceptability of:	Informing a future RCT
Intervention content & delivery	Intervention content and modes of delivery finalised
Participant recruitment	An optimal participant recruitment strategy identified
Training requirements	Research, facilitator and older adult training requirements determined
Standard operating procedures (SOPs)	SOPs developed for all study processes
Data collection tools (quantitative & qualitative)	Quantitative secondary outcome measurement tools, economic data collection tools and qualitative interview guides confirmed
Fidelity assessment procedures	Fidelity assessment procedures finalised
FITBIT	Feasibility and acceptability of using Fitbit with people with dementia confirmed
RCT protocol	RCT protocol developed

### Adverse events

In this study, the definition of an adverse event will be similar to that used in the PRINCE study, i.e. ‘Any acute alteration in the patient’s physiological condition’ [86]. It is not anticipated that participants will be at risk of experiencing an adverse event, and participants with dementia will be required to confirm that their GP has been informed and given them permission to participate in the study. Furthermore, the physical exercise programme will be individualised and tailored to each individual participant based on an exercise assessment undertaken by the physical exercise specialist and his/her ongoing weekly monitoring of participants. However, in the unlikely event that an adverse event occurs, this will be recorded by facilitators or members of the research team on an Adverse Event Reporting Form and reported to the participant’s GP, as all clinical responsibility rests with those providing routine clinical care.

### Health economic analysis

A preliminary health economic assessment of the intervention will be undertaken. The health care resources consumed will reflect the costs of organising and operating the intervention. These costs will reflect the time input of health professionals and fixed or overhead costs, including any new equipment, training, and capital expenditure related to the intervention. The feasibility and acceptability of collecting data on participants’ attendance at their general practice, hospital admissions attendances (both outpatient clinic and emergency room visits) and drug prescriptions as well as any personal expenses incurred in relation to the intervention will also be explored. Economic data will be collected for the month prior to the commencement of the intervention (baseline data) and then during the 15 weeks of the intervention. Exploring the feasibility and acceptability of collecting economic data and the appropriateness of the data collection instruments will inform the design of the economic component of any future trial.

### Social marketing

Social marketing will be used in this study to generate a better understanding of the dynamics at work in relation to stigma and dementia at community level and to map out a route toward stigma change and promote positive action for dementia. Social marketing seeks to develop and integrate marketing concepts with other methods to encourage behaviour change for the benefit of individuals and communities for the greater social good [87]. This process will begin by identifying the barriers and enablers to stigma change from the literature as well as also eliciting the views of people with dementia and caregivers regarding the barriers and enablers for stigma change. The data generated from this analysis will then be categorised and rank ordered by a multidisciplinary core modelling group led by a social marketing expert, to generate a systems map which will model visually any blockages and/or underlying dynamics that affect stigma change in relation to dementia, at a community level. The systems map will also be informed by the quantitative data from the SIS and DAS secondary measurement tools (Table 2). The final stage will involve a focus group interview with local key dementia experts to capture their perspectives and input into the systems map. This will result in the refinement of the map and the identification of leverage points to promote stigma change and help highlight where the focus should be in any subsequent study in relation to promoting stigma change for dementia.

### Sample size

**Fig. 3:**
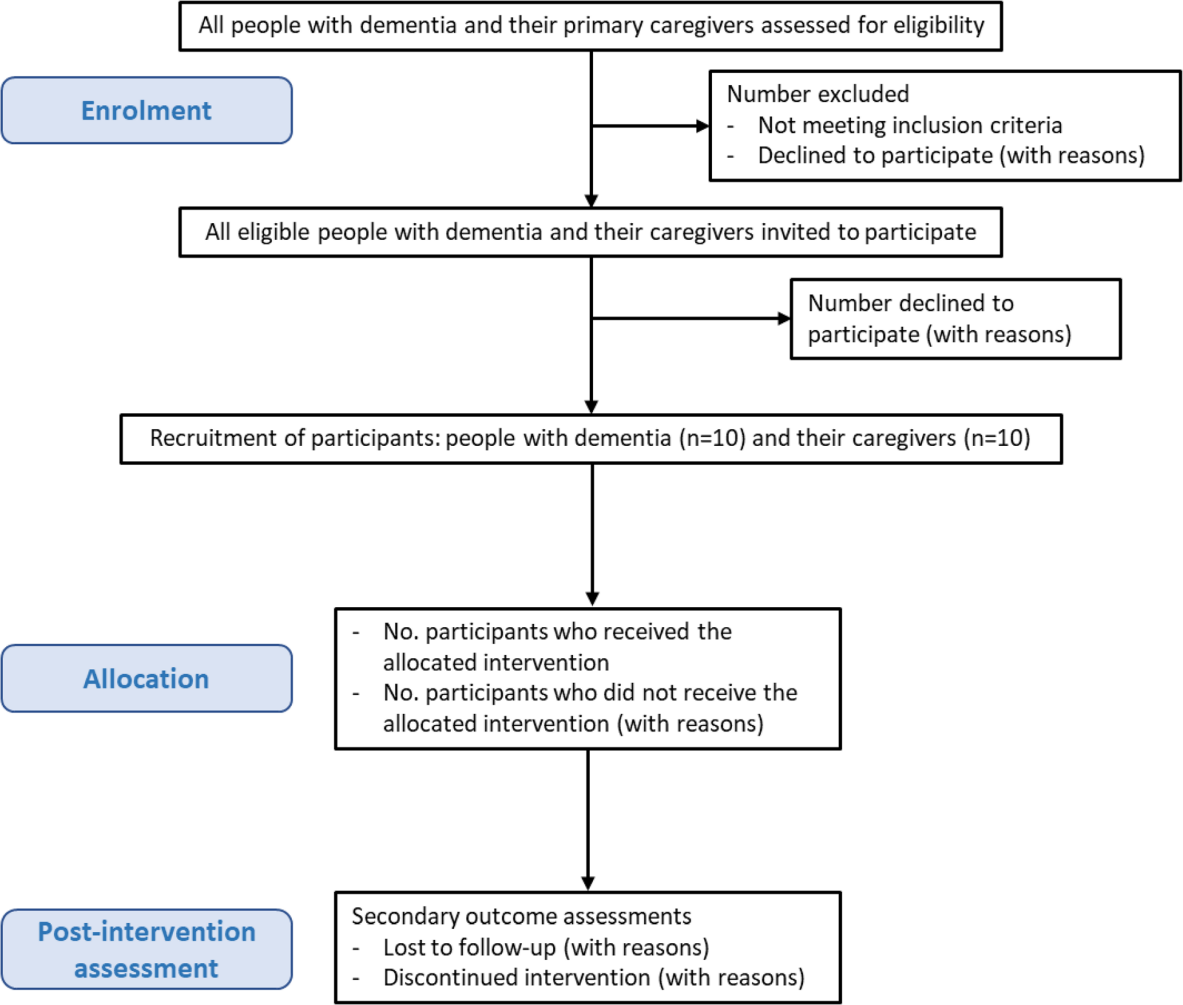
Flow of participants through the study

As this is a non-randomised feasibility study, a formal sample size calculation is not required [88]. The aim is to recruit a purposive sample of 10 people with mild to moderate dementia and their primary caregivers (*n* = 10) (20 participants in total), who meet the inclusion criteria, to participate in the CREST intervention. The flow of participants through the study is presented in Fig. 3.

### Recruitment

The initial recruitment plan involved creating a list of GP practices from the HRB Primary Care CTNI within (i) a 30-km radius of the city centre, (ii) having a population of not less than 2500 and (iii) having at least one whole time equivalent practice nurse. These practices were then contacted and invited to respond to an expression of interest regarding participating in the pilot study and to be contacted by a member of the research team. The latter inclusion criteria (ii and iii) were based on the optimal recruitment strategies used in the PRINCE study [60].

GP practices expressing an interest in taking part were asked to contact potential participants with dementia and their primary caregiver (i) informing them of the study, (ii) seeking their consent to participate and (iii) including consent to be contacted by a member of the research team. Participants willing to participate were then to be invited to meet the research team, where consent would be reconfirmed, and participants screened to confirm eligibility and baseline and feasibility data collected using the tools outlined in Table 2. Over a 10-week period, this recruitment strategy yielded only two GP practices out of 14 eligible practices. After another 8-week period, these practices were able to identify 17 participants with dementia who were eligible to participate and consented to be contacted by the research team. Of these, five participants with dementia and their caregivers consented to participate. However, two people with dementia and their caregivers subsequently withdrew, and the remaining six participants (three people with dementia and their caregivers) were retained within the study but an alternative recruitment strategy was then devised as outlined below.

The revised recruitment strategy involves contacting the gatekeepers of local dementia advocacy and health care organisations to identify any potential people with dementia and their caregivers who might be interested in participating in the CREST study. This will include The Alzheimer Society of Ireland, Western Alzheimer’s and HSE carer’s organisations. The same steps used when recruiting the GP’s will be implemented (i) informing potential participants about the study, (ii) seeking their consent to participate and (iii) including consent to be contacted by a member of the research team. Participants willing to participate will then be invited to meet the research team, where consent will be reconfirmed, including permission to contact the participants GP to confirm their eligibility to participate. Baseline and feasibility data will then be collected using the tools outlined in Table 2.

Older adults (*n* = 10) in the community will be recruited to support the physical exercise element of the intervention using a variety of recruitment methods. These will include, for example, information posters in GP practices, pharmacies, active ageing/retirement groups, older people organisations and support charities, for example, Enable Ireland and St. Vincent de Paul.

Informed consent will be obtained from all participants in the study and process consent, which enables participants to have a collaborative role in the decisions regarding their ongoing participation [89], will be utilised throughout. Older adults, people with dementia and caregivers will all receive €20 toward travel expenses to facilitate meeting the researchers during recruitment, at each data collection point in the study, as well as when attending each respective session of the intervention.

### Criterion for progression to a definitive randomised controlled trial

Decision-making about progression to a future definitive randomised controlled trial will be based on achieving the criteria for each of the study feasibility outcomes which are outlined in Table 4.

**Table 4:** CREST feasibility outcomes and the criteria for progressing to a future definitive randomised controlled trial

CREST feasibility outcomes		Criteria for determining success of feasibility to proceed to a future definitive randomised controlled trial
1	Number of participants (people with dementia and caregivers) who are screened, judged eligible and agree to take part in the study	30% of eligible participants (people with dementia & caregivers) can be recruited
2	Identification of optimal strategy for recruitment of participants for future definitive trial	Optimal recruitment strategy identified, informed by recruitment rates and qualitative data from key stakeholders
3	Identification of barriers and enablers to stigma change in dementia	A list of barriers and enablers to stigma change in dementia generated from the collection and analysis of the qualitative data from key stakeholders
4	Willingness of key gatekeepers (i.e., GPs, local Alzheimer Cafés, Western Alzheimer groups) to recruit participants	> 60% of key gatekeepers approached willing to support the recruitment of participants
5	Feasibility and acceptability of the intervention content, delivery and fidelity assessments	> 70% of participants attend ≥ 60% of intervention sessions. The intervention can be delivered in line with feasibility and fidelity targets (i.e. the content, frequency, and quality of the programmes are delivered in line with the programme manuals)
6	Follow-up rates, outcome completion and adherence or compliance rates	< 20% of participants lost to follow-up; ≥ 50% of people with dementia and caregivers adhere to the intervention
7	Reasons for non-recruitment, non-adherence or attrition	A list of the reasons for non-recruitment non-adherence or attrition generated from the qualitative data obtained from participants
8	Acceptability of the recruitment process, assessments, data collection tools, intervention content and delivery to participants	Recruitment processes, assessment, data collection tools, intervention content and delivery perceived as acceptable by > 70% of participants (people with dementia and caregivers)
9	Baseline score and variability of secondary outcome measures among participants to inform sample size estimates for a future definitive trial	Sample size estimates for required number of persons with dementia can be calculated, from baseline scores on the primary outcome measure of quality-of-life in people with Alzheimer’s disease (the QoL-AD). Variability of secondary outcomes to fall within acceptable parameters, when calculating sample size and possible attrition
10	Evaluation of cost analyses process	Economic data can be collected to inform the design of the economic component of any future trial.

*QoL-AD* Quality of Life-Alzheimer’s disease (care recipient version)

### Data management

The data management process will comply with the General Data Protection Regulation [90] requirements. All participants will be assigned a study number. The master list of participants’ names with numeric identifiers will be stored securely away from all other data in a locked filing cabinet with access available only to the members of the research team. All personal data stored on a computer will also be encrypted and password-protected in accordance with the General Data Protection Regulation (GDPR) and NUI Galway policies and procedures. Data preparation and cleaning will be conducted prior to data analysis, and any potential identifiers in the qualitative transcripts, e.g. use of names during interview, will be removed. Scores for the questionnaires will be double-checked by two researchers to confirm accuracy prior to data entry. Two researchers will also complete a visual record check of the inputted data.

### Data analysis

#### Quantitative analysis

All the study questionnaires will be inputted into the SPSS data builder to create a project database. The data can be exported to the software platform (e.g. R (3.5.2) or SPSS (V 26.0) for data cleaning and analysis. Suitable numerical and graphical summaries will be generated for demographic characteristics of the participants (e.g. percentages, measure of central tendency (means or medians), measures of variation (standard deviations or ranges). Statistical analyses appropriate for modelling changes score (e.g. ANCOVA and linear mixed models) will be carried out to estimate changes in the outcome measures from baseline to follow-up. All analyses will adhere to statistical best practice and reproducibility principles.

#### Qualitative analysis

Interviews will be recorded and transcribed verbatim, and all participants will be assigned a unique study identifier. Directed qualitative content analysis, based on the work of Hsieh and Shannon [90], will be used to analyse the data, whereby initial coding starts with a theory or relevant research findings. Researchers will then immerse themselves in the data and allow themes to emerge. Initially, each transcript will be read and then open-coded. Each code will be examined and similar codes will be broken down to form categories that are more inclusive. This will lead to a hierarchical structure of categories and subcategories. The final categories will then be arranged into groups ([90], p.1279) that best describe the data. The computer software package NVivo (QSR International Pty Ltd., Version 12, 2018) which will be used to facilitate the analysis and strategies to enhance rigour as outlined by Lincoln & Guba [91] will be put in place. The findings from the qualitative data will be used to improve the intervention and all study procedures and will inform the decision-making in relation to progressing to any future trial.

### Fidelity

Treatment fidelity assessment procedures (quality control of the staff and intervention) will be piloted to provide information on the feasibility of intervention implementation, as well as acceptability and usefulness of treatment fidelity assessment procedures. To maximise treatment fidelity, facilitators/co-facilitators delivering intervention components will be required to complete an adherence to intervention delivery form after the delivery of the components of the intervention. Participants will also be asked to complete an adherence form. An action plan will be drawn up and implemented if a component is not being delivered to the required standard.

### Trial management

A steering group will be established and will consist of key experts including dementia experts, an exercise specialist, psychologists, a trialist, a statistician, a GP, and educationalists. They will provide overall supervision of the trial and help ensure that the trial is conducted rigorously. They will also comment on and approve project documents, research approaches, and findings. In addition, they will review any adverse reports.

### Public and patient involvement (PPI)

Public and patient involvement (PPI) can be described along a spectrum from no involvement (traditional way research has been undertaken) to full partnership. We will establish a Dementia Advisory Forum consisting of people with dementia, caregivers and representatives from various other key stakeholder groups including the Alzheimer Society of Ireland, Western Alzheimers and the HSE carers groups. This advisory forum will be asked for input and advice in reviewing and providing guidance on, for example, study plans, intervention components, content and timing of delivery, data collection methods, interview guides and study results. This will help ensure that the voices and perspectives of targeted end users are heard and are incorporated into the study. PPI involvement will help us to address any issues directly that affect participants, will improve study documents and materials, and will support the dissemination and sharing of achievements in ways that promote accessibility to people with dementia living in the community and their caregivers.

### Ethics

Ethical approval has been obtained from the University Research Ethics committee (Ref: 16-Feb-03; Amend 1907, approval, August 1^st^ 2019). In addition, the university data protection officer has confirmed that the study materials and plans meet the new EU General Data Protection Regulation [92]. We will also comply with simple and clear communication guidelines as recommended by the National Adult Literacy Agency [93] and the HSE [94]. An ethical protocol will also be put in place to ensure that participants’ involvement is managed sensitively and without causing upset. In the event of modifications to the protocol, the trial register and the ethics committee will be informed and updates will be made.

## Discussion

Living well with dementia requires the implementation of multifaceted psychosocial resilience- building solutions so that people with dementia can live well for longer within their own communities. This non-randomised feasibility study will determine the feasibility of such an intervention. The results will be disseminated in peer-reviewed journals and at national and international conferences and will be used to inform the development and implementation of a subsequent RCT, should the findings support feasibility.

### Study status

Recruitment commenced 21 February 2019 and will end on 14 October 2019.

## Supplementary information

**Additional file 1.** SPIRIT 2013 Checklist: Recommended items to address in a clinical trial protocol and related documents

## Data Availability

Data sharing is not applicable to this article as no datasets were generated or analysed during the current study.

## Abbreviations

ANCOVA: Analysis of covariance
CORTE: Consent, maximizing Responses, Telling the story, and Ending on a positive note
SIS: Stigma Impact Scale
CREST: Comprehensive Resilience-building psychosocial Intervention
CST: Cognitive stimulation therapy
CTNI: Clinical Trials Network Ireland
DARES: A dementia education programme incorporating reminiscence therapy for staff
DAS: Dementia Attitudes Scale
DSM-IV: Diagnostic and Statistical Manual of Mental Disorders
GDPR: General Data Protection Regulation
GP: General Practitioner
HRB: Health Research Board
HSE: Health Services Executive
PPI: Public and patient involvement
PREPARED: Primary Care Education, Pathways and Research of Dementia
PRINCE: Pulmonary Rehabilitation Programme for people with Chronic Obstructive Pulmonary Disease in Primary Care
RCT: Randomised controlled trial
SPIRIT: Standard Protocol Items Recommendations for Intervention Trials
SPSS: Statistical Package for Social Sciences
